# What Does 16S rRNA Gene-Targeted Next Generation Sequencing Contribute to the Study of Infective Endocarditis in Heart-Valve Tissue?

**DOI:** 10.3390/pathogens11010034

**Published:** 2021-12-29

**Authors:** Paula Santibáñez, Concepción García-García, Aránzazu Portillo, Sonia Santibáñez, Lara García-Álvarez, María de Toro, José A. Oteo

**Affiliations:** 1 Center of Rickettsiosis and Arthropod-Borne Diseases (CRETAV), Infectious Diseases Department, San Pedro University Hospital-Center for Biomedical Research from La Rioja (CIBIR), 26006 Logroño, Spain; psantibanez@riojasalud.es (P.S.); cgarciag@riojasalud.es (C.G.-G.); ssantibanez@riojasalud.es (S.S.); lgalvarez.ext@riojasalud.es (L.G.-Á.); jaoteo@riojasalud.es (J.A.O.); 2 Genomics and Bioinformatics Core Facility, CIBIR, 26006 Logroño, Spain; mthernando@riojasalud.es

**Keywords:** infective endocarditis, metataxonomy, 16S rRNA gene, NGS, heart valves, Spain

## Abstract

Infective endocarditis (IE) is a severe and life-threatening disease. Identification of infectious etiology is essential for establishing the appropriate antimicrobial treatment and decreasing mortality. The aim of this study was to explore the potential utility of metataxonomics for improving microbiological diagnosis of IE. Here, next-generation sequencing (NGS) of the V3–V4 region of the 16S rRNA gene was performed in 27 heart valve tissues (18 natives, 5 intravascular devices, and 4 prosthetics) from 27 patients diagnosed with IE (4 of them with negative blood cultures). Metataxonomics matched with conventional diagnostic techniques in 24/27 cases (88.9%). The same bacterial family was assigned to 24 cases; the same genus, to 23 cases; and the same species, to 13 cases. In 22 of them, the etiological agent was represented by percentages > 99% of the reads and in two cases, by ~70%. *Staphylococcus aureus* was detected in a previously microbiological undiagnosed patient. Thus, microbiological diagnosis with 16S rRNA gene targeted-NGS was possible in one more sample than using traditional techniques. The remaining two patients showed no coincidence between traditional and 16S rRNA gene-targeted NGS microbiological diagnoses. In addition, 16S rRNA gene-targeted NGS allowed us to suggest coinfections that were supported by clinical data in one patient, and minority records also verified mixed infections in three cases. In our series, metataxonomics was valid for the identification of the causative agents, although more studies are needed before implementation of 16S rRNA gene-targeted NGS for the diagnosis of IE.

## 1. Introduction

Infective endocarditis (IE) is defined as an infection of a native or prosthetic cardiac valve, endocardial surface, or indwelling cardiac device [1]. Despite trends towards earlier diagnosis, pharmacotherapy, and surgical intervention, IE remains a major medical concern associated with high mortality and severe complications. In the last two decades it has been associated with an unchanged incidence of 1.7–10 cases/100,000 inhabitants and with a 13–25% in-hospital mortality, approaching 40% within the first year [2,3].

The variability in clinical presentation of IE and the importance of an early accurate diagnosis require a diagnostic strategy that integrates clinical, microbiological, and imaging findings. Based on these, the modified Duke criteria are widely used to guide the clinical classification of IE [4]. It is essential to identify the causative agent(s) for optimal patient management and to guide treatment duration and antibiotic choice. Staphylococci and streptococci cause about 80% of cases of IE. *Staphylococcus aureus* is the most common etiologic agent, accounting for about 30% of cases, followed by oral streptococci (~20%), other streptococci (~10%), enterococci (~10%), and coagulase-negative staphylococci (~10%). The remaining causes of IE are mainly the HACEK group organisms (*Haemophilus, Aggregatibacter, Cardiobacterium, Eikenella*, and *Kingella* species), *Coxiella burnetii, Bartonella* spp., and *Tropheryma whipplei*. Fungi are a rare endocarditis cause (~2%), with *Candida* spp. being the most common etiological agents within this group. Polymicrobial infections are responsible for 1% of the cases [1,2,3].

Endocarditis is an endovascular infection associated with continuous bacteremia. Then, positive blood culture (BC) remains the standard of microbiological diagnosis [5,6]. Routine BC allows recovery of almost all easy-to-cultivate agents of IE and provides viable bacteria for susceptibility testing. However, BC are negative in 2.5–31% of IE cases [3,7], leading to a challenging and delayed diagnosis with uncertain clinical consequences. The main causes of blood culture negative endocarditis (BCNE) are antibiotic treatment prior to BC collection and infection with fastidious microorganisms [2,8]. In patients with BCNE, the diagnostic strategy should consider serological testing for zoonotic agents including *C. burnetii*, *Bartonella* spp., *Brucella* spp., *Mycoplasma* spp., *Legionella* spp., and *Chlamydia* spp., according to local epidemiology [1,5,6]. Specific polymerase chain reaction (PCR) assays from blood (and from valve biopsies whenever available) should also be undertaken in order to confirm positive serological findings or to explore the causative agent if serological findings are negative (*T. whipplei* should be also screened). Moreover, broad range 16S and 18S ribosomal RNA (rRNA) gene PCR assays from blood (and valve if possible) are recommended. Demonstration of microorganisms by culture or histological examination in resected valvular tissue or embolic fragments is determinant for the diagnosis of IE [6]. However, it does not allow an early diagnosis, and those specimens are available only in 23–53% of cases in endocarditis [9]. Moreover, when available tissue is insufficient, culture should not be prioritized over more sensitive assays, such as molecular testing [10]. The role of molecular techniques in overcoming the limitations of culture-based methods in IE caused by fastidious microorganisms and in patients receiving antimicrobial treatment has been widely investigated. Broad-range 16S rRNA gene PCR assay followed by Sanger sequencing from blood has proven to be useful for etiologic diagnosis of IE, although higher specificity and sensitivity are achieved when testing infected cardiac valves [7,8,10,11].

In the last two decades, next-generation sequencing (NGS) technology has made it possible to investigate the composition of microbial populations in a single sequencing run with unprecedented resolution and throughput. Currently, two main approaches are used to examine the microbiome: targeted NGS, usually using the 16S rRNA gene as a phylogenetic target (metataxonomy), or shotgun metagenomics, where the genomes of all microorganisms present in the sample are sequenced. Both applications have been extensively used and have effectively transformed biomedical research, particularly focused on the human microbiota and its association with health and disease [12]. Continuous improvements through faster and user-friendly data analysis tools, the creation of accurate and comprehensive databases, and the reduction in costs have made NGS technology a promising tool in clinical microbiology, although issues relating to methodological standardization, reproducibility, and the quality of the results need to be addressed before being incorporated into clinical practice [13,14,15,16]. In the context of IE, rapid pathogen identification directly from clinical samples without the need for culturing methods promised by NGS would be a great advance for the diagnosis. Therefore, the aim of this work was to evaluate the contribution of 16S rRNA gene-targeted NGS in heart valve tissues to the study of IE.

## 2. Results

The 16S rRNA gene-targeted NGS successfully revealed bacterial pathogens in all samples of the cohort (Table 1). A total of 11,599,552 reads were obtained from Illumina MiSeq sequencing. After cleaning adapters and low-quality regions, a total amount of 9,059,264 fragments were reconstructed. Last, 8,733,978 reconstructed fragments (average counts per sample 323,481; minimum: 67,043 and maximum: 672,563), distributed among 118 Operational Taxonomic Units (OTUs) were finally assigned (NCBI-SRA: Bioproject accession number PRJNA701379).

The metataxonomic analysis of bacteria in heart valve tissues revealed the same microbiological diagnosis as conventional techniques (BC and/or valve PCR) in 24 out of 27 cases. However, the accuracy of the 16S rRNA gene-targeted NGS did not enable us to give all these results at species level. With this technology, it was possible to assign the same bacterial family as with traditional techniques for 24 cases, the same genus for 23 cases, and the same species for 13 cases. The 16S rRNA gene-targeted NGS allowed us to retrospectively confirm the previous diagnosis for all of these 24 patients. In addition, for instance, it is worth noting that findings obtained with traditional molecular techniques for patient IDs #17 and #18 were also observed when metataxonomics was performed. Thus, the metataxonomic results from patient ID #17 showed high relative abundance of *Streptococcus agalactiae* (99.9%) and low relative abundance of *Coxiellaceae* (<1%). These data corresponded to a patient diagnosed with IE by *S. agalactiae* according to BC and valve PCR, with *C. burnetii* phase II IgG titer of 400 and phase I IgG not detected, with negative PCR results for *C. burnetii*, and positive PCRs for bacteria within the *Coxiellaceae* family. Patient ID #18 was diagnosed with IE by viridans group streptococci based on traditional techniques (BC yielded *Streptococcus intermedius*, and *Streptococcus anginosus* was detected by PCR). According to the metataxonomic analysis of this specimen, the percentage of sequencing reads observed matching *S. anginosus* reached 99.7%, and other streptococci including *Streptococcus sanguinis, Streptococcus cristatus*, and *Streptococcus mutans* were found with low relative abundance (<1%).

In addition, within the clinical and epidemiologic context, the 16S rRNA gene-targeted NGS led us to corroborate mixed infections when considering relatively low abundant microorganisms (patient IDs #19, #20, and #21) and to reclassify one case (patient ID #22) as mixed infection since sequencing reads of uncultured microorganisms were detected. Thus, for patient ID #19, whose initial microbiological diagnosis included *Brucella melitensis* (by BC and PCR) and *C. burnetii* (specific phase I and phase II IgG titer of 1024 detection by indirect immunofluorescence assay and specific PCR) [17], metataxonomic results showed *Brucellaceae* as the most relative abundant taxa (99.7%) and, interestingly, *C. burnetii* was also detected at low relative abundance (<1%). For patient ID #20, conventional methods (BC and valve PCR) led to an IE diagnosis due to *Enterococcus faecalis*, and the culture of the tip of the catheter revealed *Staphylococcus epidermidis*. According to the 16S rRNA gene profiling analysis, 99.6% of reads mapped to *E. faecalis*, and members of the genus *Staphylococcus* were also observed (<1%). For patient ID #21, an initial diagnosis of IE by *S. aureus* was given according to BC and PCR methods. Furthermore, culture of the resected heart valve tissue yielded *Escherichia coli*, and bacteremia by *E. faecalis* was detected by BC after surgery. The 16S rRNA gene-targeted NGS revealed that the majority of the reads (99.6%) corresponded to *S. aureus*, and *E. faecalis* was also present in a lower proportion of reads (<1%). In this case, no sequencing reads mapping to *E. coli* were obtained. For patient ID #22, diagnosed with IE by *T. whipplei* based on valve PCR results, 16S rRNA gene-targeted NGS showed *T. whipplei* as the most abundant bacteria (99.8%), and <1% of reads were assigned to *Coxiellaceae*, suggesting a mixed infection supported by serological criteria (*C. burnetii* phase I IgG titer ≥ 1600 and phase II IgG titer of 800).

For 22 out of these 24 cases that showed consistent results among 16S rRNA gene-targeted NGS data and conventional methods, the bacterial taxa with maximum representation were found in a proportion of reads that ranged from 99.1% to 99.9%. However, for two cases (patient IDs #23 and #24), around 70% of the reads confirmed previous results (*Streptococcus mutans* and *E. faecalis*, respectively), whereas reads at nearly 30% corresponded to *E. faecalis* and *Haemophylus parainfluenzae*, respectively.

For the sample (patient ID #25) in which no microbiological diagnosis had been achieved either by BC or by PCR, the analysis of metataxonomy evidenced that most reads (95.1% of relative abundance) corresponded to *S. aureus*. Thus, with our 16S rRNA gene-targeted NGS approach we were able to detect the causative agent of IE in one more sample than with the remaining methods.

In contrast, the testing by V3–V4 16S metataxonomics yielded taxonomic predictions that differed from those obtained with conventional methods for two cases. Nevertheless, according to our 16S rRNA gene-targeted NGS data, the clinical entities found with conventional techniques were also detected in these two samples, although at low relative amounts (patient IDs #26 and #27).

Metataxonomic results corresponding to the remaining 16 patients not detailed above were consistent with those obtained by traditional techniques, and 16S rRNA gene-targeted NGS did not provide additional information considered relevant for diagnosis.

Detailed information about data that support the diagnosis of the studied patients is shown in Table 1.

## 3. Discussion

IE is still a severe disease with high morbidity and prolonged hospital stay as well as very high mortality during admission and during the 1-year follow-up [3]. Therefore, techniques to reliably guide the correct antimicrobial treatment in order to achieve the sterilization of the affected tissues and decrease the mortality are needed [10]. NGS has recently emerged as a comprehensive method for exploring causative agents of infectious diseases without prior culture. Strengths and weaknesses of the traditional methods and 16S rRNA gene-targeted NGS for the diagnosis of the infective bacteria from IE patients is shown in Table 2. As it is gathered from the table, it is important to clarify the promising role of the 16S rRNA gene-targeted NGS in the context of the IE diagnosis.

Reports about metagenomic analysis for pathogen identification in heart valve tissues are scarce, and each of them consists of very few patients [18,19,20,21,22,23,24,25,26,27]. Herein, we used 16S rRNA gene-targeted NGS from heart valve tissues as an approach to the diagnosis of IE in a cohort of 27 patients.

According to our data, metataxonomics allowed the microbiological diagnosis (*S. aureus*) in patient ID #25, in which the causative agent had not been detected either by PCR or by BC. In addition, the same microbiological diagnosis was obtained using 16S rRNA gene-targeted NGS or routine techniques for 24 patients (88.9%), although at a higher taxonomical level for 11 of them. Taxonomic assignment of sequencing reads below the genus level is a challenge in metataxonomics data analysis. Only the combination of multiple hypervariable regions or the nearly complete sequence of the 16S rRNA gene gives accurate measures of taxonomic diversity [28]. Third-generation sequencing provides long-read sequences but high base-calling error rates [29]. Additionally, consensus in current NGS protocols is essential since microbiome studies are potentially biased at every methodological stage, from sampling to bioinformatic analysis [30,31].

The interpretation of the metagenomic results for IE cases has been based on considering the bacteria represented by the highest percentage of reads as the causative agent [19,23,25]. In our series, this was possible for 22 patients, with values from 99.1% to 99.9% of the reads, and in 1 patient (patient ID #25) with 95.1% of the reads. However, we found two cases (patient IDs #23 and #24) in which the most represented bacteria matched to the initial causative agent, but the proportion reached 70% of the reads, and the remaining ones corresponded to *E. faecalis* and *H. parainfluenzae*, respectively. The biological significance of reads at around 30% of relative abundance is currently unknown. Moreover, in patient ID #26, the highest proportion of reads was as low as 26.4% and mapped to *Streptococcus* spp., and other 10 bacterial taxa were detected at relative abundance > 1% (Supplementary Table S1).

One main advantage of 16S rRNA gene-targeted NGS is its capacity to classify all bacteria from a sample without intermediate culturing steps [16]. Minority findings constitute a concern for 16S rRNA gene-targeted NGS. The cutoff value indicating how many reads of a microorganism in a sample are not relevant for the analysis has not been established for microbiome studies. It will require a vast experience to establish which spurious reagent contaminants, sample processing contaminants, cross-contamination in multiplexed libraries, etc. or true infections or coinfections are present. Regarding the relatively low abundant microorganisms, we have described three cases of previously characterized mixed infections (patient IDs #19, #20, and #21) and one case reclassified as possible mixed infection in agreement with serological results (patient ID #22). However, there were two cases in which the microorganism formerly considered as causative agent by BC and/or PCR was barely represented in metataxonomic results (patient IDs #26 and #27). In order to understand this, it is important to take into consideration that, as was previously mentioned, each step of NGS analysis influences the relative abundances observed [29,30,31]. However, it cannot be discarded that these small percentages are inherent failures of the technique. The concordance of minority percentages with clinical data gave value to the diagnosis of mixed infectious in the four cases mentioned above (patient IDs #19–#22), in contrast with patient ID #27, in which no clinical or epidemiological data available supported streptococcal infection, and patient ID #26, in which 11 bacterial taxa were found. Addressing laboratory contamination is an urgent task, and it is important to scrutinize NGS data with an understanding of its potential for false positive results. Bacterial identification using 16S rRNA gene-targeted NGS may be biased because of unequal amplification of certain species, and it is influenced by several factors, such as the region(s) sequenced, amplification efficiency, sequencing technology, and bioinformatics workflow(s). In order to assure the quality of the NGS results, it is recommended to include spiking-in mock microorganisms that provide comparable results across research groups and time, as well as positive and negative controls [29,31].

NGS technology has been suggested as an essential supplement to culture-based methods for the diagnosis of IE, particularly when the causative agent does not grow [18,19,24,25]. In our experience, for one BCNE case (patient ID #25) in which BC and conventional PCR had failed, 16S rRNA gene-targeted NGS allowed us to point to *S. aureus* as the causative agent, while in three more BCNE cases from this study, the results were in concordance with the microbiological diagnosis already achieved by PCR (patient IDs #1, #11, and #22). Moreover, the application of NGS technology using whole genome sequencing of bacteria related to IE after BC isolation has recently allowed the characterization of emerging microorganisms associated with this entity (e.g., *Bergeyella cardium*) as well as the description of new mutations related to antibiotic resistance in *E. faecalis* strains, suggesting the occurrence of new antibiotic resistance mechanisms [32,33]. When applied to valve tissue, NGS may provide relevant information about therapeutic options after cardiac surgery for IE patients, especially for BCNE. Whereas the 16S rRNA gene-targeted NGS only allows detection of bacteria, metagenomic NGS can also identify fungi and viruses. However, the clinical utility of these approaches remains uncertain since clinically irrelevant microorganisms may be detected. Even though the application of NGS techniques may not always be valuable, and considering that the techniques are more expensive and time-consuming and that they require equipment hardly affordable by most clinical laboratories and personnel trained in bioinformatics, this study is so far one of the largest published series, and the concordance of our results with the previous microbiological diagnoses in almost all patients highlights the importance of this work.

## 4. Materials and Methods

### 4.1. Samples

DNA from resected heart valves extracted using QIAamp DNA Mini Kit (Qiagen, Hilden, Germany) and stored at −80 °C were retrospectively selected from the ‘Zoonosis collection’ registered in the National Registry of Biobanks of the Carlos III Health Institute (Reference: C.0006409), located in CRETAV (CIBIR, La Rioja, Spain). They corresponded to 27 patients diagnosed with IE according to the modified Duke criteria in our hospital (Hospital Universitario San Pedro, La Rioja, Spain) from 2009 to 2017. Main epidemiological and clinical characteristics are shown in Table 3. Microbiological data including BC and 16S rRNA gene PCR [34] and Sanger sequencing results from the heart valve tissues were available in all cases (Table 1).

Approval of the regional ethics committee was obtained (Comité Ético de Investigación Clínica-Consejería de Sanidad de La Rioja, Ref. CEICLAR PI-19). Informed consent was obtained from all participants. All procedures were in accordance with the ethical standards of the research committee and with the 1964 Helsinki Declaration and its later amendments.

### 4.2. DNA Quantification and Quality Determination

DNA was quantified with a Qubit 3.0 fluorometer (Thermo Fisher Scientific, Waltham, MA, USA) using Qubit dsDNA HS (High Sensitivity) assay kit. The quality of DNA was assessed with the Fragment Analyzer (Agilent, Santa Clara, CA, USA), using Genomic DNA 50 kb kit. A total of 12.5 ng DNA per sample were added.

Samples had been manipulated under sterile conditions in a Class II biosafety cabinet using cycles of UV light prior and between uses to prevent contamination. Sterile single-use instruments were used. DNA extraction, preparation of PCR master mix, and amplification had been performed in separate rooms to prevent contamination. All the kit reagents were previously tested for the absence of microorganisms using 16S rRNA gene PCR [34]. Moreover, negative controls of extraction (blanks) corresponding to extraction tubes without valve biopsy specimens were included in parallel.

### 4.3. 16S rRNA Gene Amplification, Library Preparation, and Sequencing

Primers targeting the hypervariable V3–V4 regions of 16S rRNA gene were used [35]. Negative controls were included in the PCR assays. Amplified regions were purified and indexed with Nextera XT Index kit (Illumina, San Diego, CA, USA). The library quality was assessed on a Qubit 3.0 Fluorometer and Fragment Analyzer using a dsDNA reagent (35–5000 bp) kit. Paired-end 300 bp sequences were obtained on an Illumina MiSeq platform.

### 4.4. Sequence Processing and Analysis

Quality controls of raw reads were carried out with FastQC software <http://www.bioinformatics.babraham.ac.uk/projects/fastqc> (accessed on 14 December 2021) [36] and trimmed with Trimmomatic software [37]. The V3–V4 amplified region (550–580 bp) was reconstructed through paired reads according to the Quantitative Insights Into Microbial Ecology (QIIME) protocol (v1.9.1) [38]. OTUs were defined as sequences with at least 97% similarity versus the Greengenes database [39] using the UClust clustering algorithm <http://drive5.com/usearch/> (accessed on 14 December 2021) [40] and following the open-reference method described by QIIME [41]. OTUs with <0.01% relative abundance of the total read counts on a per-sample basis were removed (spurious and chimeric reads). Each OTU’s sequences were refined with the BLAST tool against GenBank <https://blast.ncbi.nlm.nih.gov/Blast.cgi?PROGRAM=blastn&PAGE_TYPE=BlastSearch&LINK_LOC=blasthome#> (accessed on 14 December 2021) [42]. The causative pathogen was attributed to the microorganism with which the most amplicon reads matched. Low-abundant taxa were also considered relevant when a diagnosis with mixed infections matched epidemiologic, clinical, and microbiological results of patients.

## 5. Conclusions

Results of 16S rRNA gene-targeted NGS are mostly consistent with those of BC and/or conventional PCR but do not improve the diagnosis of IE cases. Metataxonomics may be helpful to IE patients after valve replacement surgery, especially when conventional tests fail to yield a diagnosis. Moreover, minority findings supported by clinical data could suggest mixed infections not previously suspected, although more efforts should be made in order to understand them. Hence, further studies are required to validate the clinical usefulness of this method.

## 6. Addendum

From the design of this study to the present time, a new version of QIIME was published (qiime2-2020.8). That is why, for a more in-depth exploration of the use of amplicon sequencing in the context of IE, bacterial taxa with the highest relative abundance using different bioinformatics pipelines are compared in Table 4. According to our data, the newest version of QIIME allowed us to increase the species level accuracy in two samples (patient IDs #8 and #17).

The same reads (Fastq_raw, obtained directly from the Illumina MiSeq sequencing machine) were reanalyzed by qiime2-2020.8. DADA2 was used for denoising and for obtaining Amplicon Sequence Variants (ASVs). Additionally, the taxonomic assignment was made by SILVA (version 132, released on 10 April 2018) since it is more updated than Greengenes (not updated since 2013). In order to improve the taxonomic assignment of ASVs, SILVA was trained in the V3–V4 region of 16S rRNA gene, which was our target.

## Figures and Tables

**Table 1:** Contributions of 16S rRNA gene-targeted NGS from heart valve tissue specimens to the diagnosis of infective endocarditis for the 27 patients of this study.

	Patient ID	Initial Microbiological Diagnosis before 16S rRNA Gene-Targeted NGS	Identification Technique(s) for Diagnosis	Bacterial Taxa with the Highest Relative Abundance and Minority Findings Suspicious of Being Clinically Important by 16S rRNA Gene-Targeted NGS
High confident consistent group	#1	*Staphylococcus aureus* ^1^	PCR, WEC ^1^	*Staphylococcus* spp. (99.9%)
	#2	*Staphylococcus epidermidis*	BC, PCR	*Staphylococcus* spp. (99.8%)
	#3	*Staphylococcus lugdunensis*	BC, PCR	*Staphylococcus* spp. (99.9%)
	#4	*Streptococcus bovis* group	BC, PCR	*Streptococcus* spp. (99.1%)
	#5	*S. bovis* group	BC, PCR	*Streptococcus* spp. (99.1%)
	#6	*Streptococcus milleri*	BC	*Streptococcus* spp. (99.6%)
	#7	*Streptococcus mitis*	BC, PCR	*Streptococcus* spp. (99.8%)
	#8	*Streptococus sanguinis*	BC, PCR	*Streptococcus* spp. (99.8%)
	#9	*S. mitis*	BC, PCR	*Streptococcus* spp. (99.9%)
	#10	*Streptococcus oralis*	BC, PCR	*Streptococcus* spp. (99.9%)
	#11	*Coxiella burnetii*	PCR	*C. burnetii* (99.5%)
	#12	*Enterococcus faecalis*	BC, PCR	*E. faecalis* (99.9%)
	#13	*E. faecalis*	BC, PCR	*E. faecalis* (99.8%)
	#14	*E. faecalis*	BC, PCR	*E. faecalis* (99.9%)
	#15	*E. faecalis*	BC, PCR	*E. faecalis* (99.7%)
	#16	*Haemophylus parainfluenzae*	BC, PCR	*H. parainfluenzae* (99.5%)
	#17	*Streptococcus agalactiae*	BC, PCR	*S. agalactiae* (99.9%), *Coxiellaceae* (<1%)
	#18	*Streptococcus anginosus* group (*Streptococcus intermedius* ^2^*, S. anginosus* ^3^)	BC ^2^, PCR ^3^	*S. anginosus* (99.7%), *Streptococcus* spp. (<1%)
Corroborated mixed infections	#19	*Brucella melitensis,* *C. burnetii* ^4^	BC, PCR, IFA ^4^, *htpAB* PCR ^4^	*Brucellaceae* (99.7%), *C. burnetii* (<1%)
	#20	*E. faecalis, S. epidermidis* ^5^	BC, PCR, CTC ^5^	*E. faecalis* (99.6%), *Staphylococcus* spp. (<1%)
	#21	*S. aureus, Escherichia coli* ^6^ *, E. faecalis* ^7^	BC, PCR, VC ^6^, BC ^7†^	*S. aureus* (99.6%), *E. faecalis* (<1%)
Reclassified as mixed infection	#22	*Tropheryma whipplei*	PCR	*T. whipplei* (99.8%), *C. burnetii* (<1%)
Low confident consistent group	#23	*E. faecalis*	BC, PCR	*E. faecalis* (68.8%), *H. parainfluenzae* (29.7%)
	#24	*Streptococcus mutans*	BC, PCR	*S. mutans* (69.5%), *E. faecalis* (28.6%)
New diagnosis	#25	No etiology	BC, PCR	*S. aureus* (95.1%)
Discordant diagnosis	#26	*E. faecalis*	BC, PCR	*Streptococcus* spp. (26.4%), *E. faecalis* (15.9%) *
	#27	*H. parainfluenzae*	BC	*Streptococcus* spp. (99.2%), *H. parainfluenzae* (<1%)

ID, identification number; 1, 2, 3, 4, 5, 6 and 7, technique by which the microorganism was detected; PCR, polymerase chain reaction (targeting 16S rRNA gene from heart valve tissues); WEC, wound exudate culture; BC, blood culture; IFA, immunofluorescence assay; *htpAB* PCR, PCR targeting the *htpAB* gene for *C. burnetii*; CTC, catheter tip culture; VC, heart valve culture; ^†^, after surgery. *, a total of 11 bacterial taxa were detected at relative abundance > 1% (See Supplementary Table S1).

**Table 2:** Main advantages and disadvantages of classic techniques and 16S rRNA gene-targeted NGS for the microbiological diagnosis of infected endocarditis.

Technique	Advantages	Disadvantages
Blood culture	Cornerstone of diagnosis Bacterial identification and susceptibility testing Simple collection procedure Faster if associated with MALDI-TOF Available for clinical microbiology labs	Limited sensitivity, especially after antibiotic therapy or for fastidious microorganisms Delayed diagnosis if negative Processing time: several days
		
PCR	More sensitive and faster than culture Applicable for blood and valve tissue (variable collection procedure) Can be broad range or specifically targeted (high specificity) Especially useful for BCNE Processing time: several hours (<1 day)	Variable sensitivity (blood vs. valve; 16S rRNA gene vs. specific targets) Requires careful clinical correlation (detection of viable and non-viable organisms, risk of contamination) Not available for all clinical microbiology labs
		
Valve culture	Definitive diagnosis Bacterial identification and susceptibility testing Available for clinical microbiology labs	Low specificity (tedious handling of sample) Limited sensitivity, especially after antibiotic therapy or for fastidious microorganisms Difficulty for sample acquisition (surgery) Delayed diagnosis Processing time: several days
		
Serology	Particularly useful in BCNE caused by *Coxiella burnetii*, *Bartonella* spp., and other fastidious microorganisms Simple collection procedure Processing time: two hours	Low sensitivity and specificity High seroprevalence in certain collectives for *C. burnetii* and *Bartonella* spp. High titers can persist and require careful clinical correlation
		
16S rRNA gene-targeted NGS	High-throughput sequencing Detection of all bacteria present in a sample Culture independent Promising diagnostic tool	Variable sensitivity (targeted region, bioinformatics pipeline, equipment, etc.) Lack of consensus for processing and data analysis Bioinformatics skills and computational resources are needed Requires careful clinical correlation (detection of viable and non-viable organisms, risk of contamination) Processing is time-consuming

MALDI-TOF, matrix-assisted laser desorption/ionization-time-of-flight; PCR, polymerase chain reaction; BCNE, blood culture-negative endocarditis; NGS, next-generation sequencing.

**Table 3:** Main epidemiological and clinical characteristics of the 27 patients.

ID	Year of Diagnosis	Age, Sex	Affected Tissue	IE Definition *	Cardiac History	Most relevant Historical Conditions	Charlson Comorbidity Index ^†^	Vegetation	Fever	Embolisms	Heart Murmur	Vascular Phenomena	Intracardiac Complications	Cardiac Failure	Antibiotic Therapy (Days) ^^^	Mortality
#1	2012	56, F	ivd	P	CD, AVB, PMC	-	2	N	Y	N	N	N	N	N	1	N
#2	2013	69, M	pav	P	CD, AS, MI, HS	-	3	Y	Y	N	Y	N	Y	N	6	UN
#3	2017	67, M	nav	D	CD, AI, AF	ARF	4	Y	N	N	N	N	Y	Y	11	N
#4	2011	64, M	ivd	D	CD, HF, DM, AF	LD	8	Y	Y	N	N	N	N	N	48	N
#5	2012	71, F	nav	D	-	-	3	Y	Y	N	Y	N	N	Y	6	Y
#6	2014	68, F	pmv	D	AF, TI, NVD	n-aHC	7	Y	Y	N	N	N	Y	N	18	Y
#7	2016	53, M	nav	D	-	-	1	Y	Y	N	Y	N	Y	N	8	N
#8	2016	54, F	nmv	D	-	HP	1	Y	Y	N	Y	N	N	N	22	N
#9	2016	53, M	nmv	D	CD	CLD	2	Y	N	Y	Y	N	N	N	18	N
#10	2017	45, M	nmv	D	-	-	0	Y	Y	N	Y	N	N	N	13	N
#11	2011	UN, F	ivd	UN	UN	UN	UN	UN	UN	UN	UN	UN	UN	UN	UN	UN
#12	2015	69, F	nmv	D	-	GBS	3	Y	Y	Y	N	Y	Y	N	50	N
#13	2015	75, M	nav	D	-	n-aAC	3	Y	Y	Y	Y	N	N	N	17	N
#14	2015	75, M	nmv	D	-	-	3	Y	Y	N	Y	N	Y	Y	22	N
#15	2017	75, M	pav	D	CHF, CD	CLD	6	Y	Y	N	Y	N	Y	N	14	N
#16	2017	26, F	nmv	D	CHD, MI	-	0	Y	Y	N	Y	N	N	N	27	N
#17	2013	45, M	nmv	D	VH, MI	n-aAC, HH	1	Y	Y	N	Y	N	N	Y	15	Y
#18	2015	48, M	nmv	D	MI	-	0	Y	Y	Y	N	Y	N	N	13	N
#19	2009	51, M	ivd	UN	UN	UN	UN	Y	Y	UN	UN	Y	UN	UN	UN	N
#20	2010	80, M	nav	D	AF, CD, NVD	n-aPC, ARF	8	Y	Y	N	N	N	N	N	17	N
#21	2014	70, M	nav	D	AF	ITP	3	Y	N	N	N	N	Y	Y	12	Y
#22	2014	48, M	nav	D	-	-	0	Y	Y	N	N	N	N	Y	2	N
#23	2017	84, M	pav	D	-	PVD	5	Y	N	N	Y	N	Y	N	11	N
#24	2015	56, F	nmv	D	NVD	GBD, HYF, FLD	4	Y	Y	Y	N	N	Y	N	11	N
#25	2011	26, M	nmv	D	CHD, VD, WPWS	-	0	Y	Y	N	Y	N	Y	N	5	N
#26	2013	76, M	nmv	D	NVD, AF	COPD, CVDn-aPC	7	Y	N	Y	N	N	N	N	11	N
#27	2015	17, F	ivd	D	CHD	-	0	Y	Y	N	N	N	N	N	12	N

*, according to the modified Duke criteria; ^†^, age-adjusted; ^, days on effective antibiotic therapy prior to valve resection; F, female; ivd, intravascular device; P, possible; CD: coronary disease; AVB, atrioventricular block; PMC, pacemaker carrier; -, not relevant information; N, no; Y, yes; M, male; pav, prosthetic aortic valve; AS, aortic stenosis; MI, mitral insufficiency; HS, heart surgery; UN, unavailable; nav, native aortic valve; D, definite; AI, aortic insufficiency; AF, atrial fibrillation; ARF, acute renal failure; HF, heart failure; DM, dilated cardiomyopathy; LD, liver disease; pmv, prosthetic mitral valve; TI, tricuspid insufficiency; NVD, natural valvular disease; n-a HC, non-active hepatocarcinoma; nmv, native mitral valve; HP, hip prosthesis; CLD, chronic lung disease; GBS, Guillain-Barre syndrome; n-a AC, non-active adenocarcinoma; CHF, congestive heart failure; CHD, congenital heart disease; VH, ventricular hypertrophy; HH, hepatic hemangiomas; n-a PC, non-active prostate carcinoma; ITP, immune thrombocytopenic purpura; PVD, peripheral vascular disease; GBD, Graves–Basedow disease; HYF, hyperferritinemia; FLD, fatty liver disease; VD, valvular disease; WPWS, Wolff–Parkinson–White syndrome; COPD, chronic obstructive pulmonary disease; CVD, cerebrovascular disease.

**Table 4:** Comparison between the results obtained by different bioinformatics pipelines.

Patient ID	Initial Diagnosis	QIIME 1 ^1^		QIIME 2 ^2^		Data Refined with BLAST	
#1	*Staphylococcus aureus*	78.7%	*Planococcaceae*	99.9%	*Staphylococcus* spp.	99.9%	*Staphylococcus* spp.
#2	*Staphylococcus epidermidis*	97.5%	*Planococcaceae*	98.4%	*Staphylococcus* spp.	99.8%	*Staphylococcus* spp.
#3	*Staphylococcus lugdunensis*	97.1%	*Planococcaceae*	99.8%	*Staphylococcus* spp.	99.9%	*Staphylococcus* spp.
#4	*Streptococcus bovis* group	99.9%	*Streptoccocus* spp.	99.9%	*Streptococcus* spp.	99.1%	*Streptococcus* spp.
#5	*S. bovis* group	99.9%	*Streptoccocus* spp.	98.6%	*Streptoccocus* spp.	99.1%	*Streptoccocus* spp.
#6	*Streptococcus milleri*	99.8%	*Streptoccocus* spp.	99.3%	*Streptoccocus* spp.	99.6%	*Streptococcus* spp.
#7	*Streptococcus mitis*	99.9%	*Streptoccocus* spp.	99.3%	*Streptoccocus* spp.	99.8%	*Streptoccocus* spp.
#8	*Streptococcus sanguinis*	99.8%	*Streptoccocus* spp.	99.5%	*S. sanguinis*	99.8%	*Streptoccocus* spp.
#9	*S. mitis*	99.9%	*Streptoccocus* spp.	99.7%	*Streptoccocus* spp.	99.9%	*Streptoccocus* spp.
#10	*Streptococcus oralis*	99.9%	*Streptoccocus* spp.	98.8%	*Streptoccocus* spp.	99.9%	*Streptococcus* spp.
#11	*Coxiella burnetii*	99.5%	*Coxiella* spp.	98.8%	*C. burnetii*	99.5%	*C. burnetii*
#12	*Enteroccus faecalis*	99.4%	*Enterococcus* spp.	99.9%	*Enterococcus* spp.	99.9%	*E. faecalis*
#13	*E. faecalis*	99.1%	*Enterococcus* spp.	99.8%	*Enterococcus* spp.	99.8%	*E. faecalis*
#14	*E. faecalis*	99.5%	*Enterococcus* spp.	99.9%	*Enterococcus* spp.	99.9%	*E. faecalis*
#15	*E. faecalis*	98.8%	*Enterococcus* spp.	99.8%	*Enterococcus* spp.	99.7%	*E. faecalis*
#16	*Haemophilus parainfluenzae*	99.5%	*H. parainfluenzae*	88.3%	*Haemophilus* spp.	99.5%	*H. parainfluenzae*
#17	*Streptococcus agalactiae*	99.9%	*Streptoccocus* spp.	97.8%	*S. agalactiae*	99.9%	*S. agalactiae*
#18	*Streptococcus anginosus* group	99.7%	*S. anginosus*	99.6%	*S. anginosus* subsp. *anginosus*	99.7%	*S. anginosus*
#19	*Brucella melitensis*	99.7%	*Ochrobactrum* spp.	94.7%	*Ochrobactrum* spp.	99.7%	*Brucellaceae*
#20	*E. faecalis*	99.1%	*Enterococcus* spp.	99.6%	*Enterococcus* spp.	99.6%	*E. faecalis*
#21	*S. aureus*	99.6%	*S. aureus*	99.9%	*Staphylococcus* spp.	99.6%	*S. aureus*
#22	*Tropheryma whipplei*	99.8%	*Microbacteriaceae*	98.3%	*T. whipplei*	99.8%	*T. whipplei*
#23	*E. faecalis*	68.5%	*Enterococcus* spp.	62.4%	*Enterococcus* spp.	68.8%	*E. faecalis*
#24	*Streptococcus mutans*	69.8%	*Streptoccocus* spp.	71.1%	*S. mutans*	69.5%	*S. mutans*
#25	No etiology	95.1%	*S. aureus*	95.6%	*Staphylococcus* spp.	95.1%	*S. aureus*
#26	*E. faecalis*	26.9%	*Streptoccocus* spp.	25.6%	*S. sanguinis*	26.4%	*Streptoccocus* spp.
#27	*H. parainfluenzae*	99.8%	*Streptoccocus* spp.	55.5%	*S. mutans*	99.2%	*Streptoccocus* spp.

^1^, Bioinformatics pipeline based on QIIME (v1.9.1) and Greengenes (as described in Material and Methods); ^2^, Bioinformatics pipeline based on QIIME2 (qiime2-2020.8) and SILVA (as described in Addendum); subsp., subespecie.

## Data Availability

All raw read files from NGS have been submitted to the NCBI Sequence Read Archive (SRA) under Bioproject accession number PRJNA701379. Partial results were presented at VII Congreso SEICAV (Sociedad Española de Infecciones Cardiovasculares), Sevilla (Spain), 2018. The manuscript was sent to the preprint server medRxiv: https://doi.org/10.1101/2021.01.23.21250364 (accessed on 14 December 2021).

## Supplementary Materials

The following are available online at https://www.mdpi.com/article/10.3390/pathogens11010034/s1, Table S1: Bacterial taxa detected at relative abundance > 1% in patient ID #26 [43,44,45,46,47,48,49,50,51,52,53].
