# Supplementary material for: What Does 16S rRNA Gene-Targeted Next Generation Sequencing Contribute to the Study of Infective Endocarditis in Heart-Valve Tissue?
Source: Pathogens. 2021 Dec 29;11(1):34. doi: 10.3390/pathogens11010034 (PMC8781873; doi:10.3390/pathogens11010034)
Supplement: Supplementary file 1 [file pathogens-11-00034-s001.zip › pathogens-1532173-supplementary.pdf]

Supplementary data Table S1: Bacterial taxa detected at relative abundance >1% in patient ID #26.

Bacteria	IE agent	Clinical importance	Reference
Abiotrophia defectiva	<1%	Normal flora of the oral cavity, the urogenital and the intestinal tracts. It has been involved in ocular, osteoarticular, arthroplasty and sinus infections, otitis, cerebral abscesses, iatrogenic meningitis and pancreatic abscesses.	[43]
Actinomyces spp.	30 cases since 1939	Infections are usually limited to cervicofacial, thoracic, and abdominopelvic regions.	[44]
Bergeyella zoohelcum	rare	It is a well-known zoonotic pathogen causing cellulitis, leg abscess, tenosynovitis, septicemia, pneumonia and meningitis.	[33]
Capnocytophaga sputigena	yes	Normal flora of the oral cavity. It has occasionally been associated with periodontitis, sinusitis, conjunctivitis, pleural empyemas, subphrenic abscesses, osteomyelitis and septicemia.	[45]
Corynebacterium spp.	yes	Normal skin flora. It contaminates blood cultures and causes opportunistic severe infections.	[46]
Enterococcus faecalis	10% (Enterococci)	Part of the human gastrointestinal flora. It causes urinary, intra-abdominal, wound and pelvic infections and bacteremia. It is the third most common cause of IE.	[47]
Haemophilus parainfluenzae	1.2 - 3% (HACEK group)	It frequently colonizes the oropharynx and nasopharynx and may also be part of the genital tract microbiota. Related to infections of the respiratory tract although brain abscess, surgical site infections, soft tissue infections, prosthetic joint infections, and hepatic and biliary tract infections have also been documented. Most common cause of HACEK endocarditis.	[48]
Lautropia mirabilis	no	Commonly found in the human oral cavity and the upper respiratory tract. Described in HIV-positive children and in the sputum of one cystic fibrosis patient.	[49,50]
Neisseria elongata	very rare	Normal oropharyngeal bacterial flora. Associated with septicemia and osteomyelitis.	[51]
Rothia aeria	9 cases	It colonizes the human oral cavity and upper gastrointestinal tract. It is sporadically considered as cause of neck abscess, shoulder joint infection, lung infection and neonatal sepsis.	[52]
Streptococcus spp.	30%	Streptococci are the second leading cause of IE. They are found in almost every location in the human body and are the dominant species in the human oral cavity and upper respiratory tract.	[53]

HACEK, *Haemophilus* species, *Aggregatibacter* species, *Cardiobacterium hominis*, *Eikenella corrodens*, and *Kingella* species; IE, infective endocarditis.
